# The influence of social network on couples’ intention to have the first child

**Published:** 2013-03

**Authors:** Talat Khadivzadeh, Robab Latifnejad Roudsari, Masoud Bahrami, Ali Taghipour, Jalal Abbasi Shavazi

**Affiliations:** 1 *Department of Midwifery, School of Nursing and Midwifery, Isfahan University of Medical Sciences, Isfahan, Iran.*; 2 *Department of Midwifery, School of Nursing and Midwifery, Mashhad University of Medical Sciences, Mashhad, Iran.*; 3 *Department of Midwifery, School of Nursing and Midwifery, Patient safety and Health Quality Research Center, Mashhad University of Medical Sciences, Mashhad, Iran.*; 4 *Nursing and Midwifery Research Center, School of Nursing and Midwifery, Isfahan University of Medical Sciences, Isfahan, Iran.*; 5 *Health Sciences Research Center, Department of Biostatistics and Epidemiology, School of Health, Mashhad University of Medical Sciences, Mashhad, Iran.*; 6 *Department of Demography, University of Tehran, Tehran, Iran.*

**Keywords:** *Fertility*, *Childbirth*, *Social network*, *Qualitative study*, *Content analysis*

## Abstract

**Background: **Recently, the relevance of social interactions as determinants of behavioral intentions has been increasingly perceived, but there is a lack of knowledge on how and why it interacts with couples’ fertility intentions.

**Objective:** This qualitative study was conducted to explore the influence of social network on couples’ intention to have their first child in urban society of Mashhad, Iran in 2011.

**Materials and Methods: **In this exploratory qualitative study in-depth interviews were conducted with 24 participants including 14 fertile women, two parents, three husbands and five midwives and health care providers. The sample was selected purposively in urban health centers, homes and workplaces until data saturation was achieved. Data analysis was carried out adopting conventional content analysis approach through giving analytical codes and identification of categories using MAXqda software. Study rigor verified via prolonged engagement, validation of codes through member check and peer debriefing.

**Results: **Findings from data analysis demonstrated four major categories about social network’s influence on couples’ intention to have their first child including 1) perception of fertility relevant social network, 2) occurrence of various types of social influence 3) subjective judgment to the benefits of social network and its fitness to personal life, and 4) couples’ interaction with social network.

**Conclusion: **Managing the fertility behaviors need to include the consideration of personal social networks surrounding the couples. It is important to apply the study findings in providing family planning services and dissemination of appropriate fertility behaviors through community-based reproductive health care delivery system.

## Introduction

The birth of the first child of a couple is deemed as a critical event (1). Having a child is associated with many positive general values such as gaining an emotional advantage, economic benefits and security, self-enrichment and development and also the sense of cohesiveness and continuity (2). On the other hand, sometimes having a child causes struggles to adapt to parenthood and experiencing a significant decline in the couple relationship adjustment (3). 

A great number of factors including economical, social, emotional and individual issues as well as attitude towards parenthood have crucial role in fertility decisions and influence the couple’s experience of starting the childbearing (4). In recent decades the relevance of social interactions or social networks to assess fertility behaviors has been increasingly investigated (5-7). Social network includes the individuals or groups linked by some common ties that shape the availability of access to information and other resources (8-10). 

Being surrounded by a network of social relations influences couples' decisions which enables or restricts their choices (11, 12). Individuals learn, transmit, negotiate and challenge social norms in social interactions (13). Studies on fertility have paid attention to the importance of social contexts including family, peer groups and health care providers on fertility intentions (14-15). Fertility behavior is a research area in which little is known about how meaning and subjective perceptions are created in interactions with relevant others and the way that they shape individuals' behavior (6). Study of Arai on teenage pregnancies showed that imperative relationships affect fertility (16). In the study of Bernardi, social relationships had an influential role on fertility decisions, for instance, the impact of peer groups on fertility behavior in many aspects was more significant than the impact of the family (17). Some previous studies discussed the relationship between age at first childbirth in parents and their children (18-20).

In spite of substantial efforts to model the role that social factors plays in the development of fertility intentions, there is little empirical evidence about the mechanisms and patterns of such influences (21). In the recent years Iran has faced a lot of challenges in fertility management. Mashhad city with a high rate of migrants as well as having more than 85% of its population stayed in urban areas, consistent with the country, has had a great deal of evolutions in fertility indicators in recent decades (22). There is no research in Iran that addresses the issue of social network’s inﬂuence on fertility intentions. Considering that qualitative methods can make a valuable contribution to social network research (6). This qualitative study was designed to explore the patterns of inﬂuence of social networks on the individual couples’ intention for having their first child in the socio-cultural context of Iran.

## Materials and methods

**Design**

The present paper is part of a large exploratory qualitative study on couples’ fertility behavior in urban society of Mashhad, Iran, which presents the findings of a qualitative content analysis. Qualitative approach is proper to investigate new areas and topics about which little is known and allow researcher to explore participants’ experiences that have not investigated before (23). 

Qualitative approach has the ability to explore aspects of complex behaviors, attitudes and interactions that quantitative methods cannot (24). Peddie and Teijlingen have introduced qualitative methods as valuable tools in fertility and reproduction related studies (24). As emotional and psycho-social influences are crucial in fertility related issues, quantitative methods may not allow researchers to explore these issues in depth. 

**Setting**

The study setting was Family Planning Units of urban health centers, homes and workplaces in Mashhad- the second largest city of Iran- with a resident population of around 3,000,000 based on 2011 national consensus (25). 

**Sampling**

Twenty four participants including 14 married fertile women aged 15-49 years, five midwives and family health care providers, two participants’ mothers, three participants’ husbands and one physician were purposively selected to be interviewed. At first, participant were purposefully recruited from the eligible clients who attended the Family Planning Units in urban health centers in Mashhad and then sampling continued in all other settings including homes and workplaces. To be ensured of maximum variation, participants were selected from various ages, educational levels and socio-economic backgrounds in order to get diversity in experiences, perceptions and beliefs about childbearing. 

All participants were Iranian, spoke in Farsi and lived in urban district of Mashhad. The fertile women included in the study had at least one child within their current marriage and had no child from any prior marriage. The women excluded if they experienced menopause. Midwives and family health care providers worked in urban district of Mashhad. Full experienced participants were introduced by health care providers and previous participants. Participants were invited by phone call or face to face in the clinics and were asked to participate in the study. 

**Profile of participants**

The mean age of participant women was 31±6.4 years. They had their first child at age of 23±3.9 years on average. 35.7% of participants were graduates from higher education, 28.6% from secondary and 35.7% from elementary education. Most of participants were born in Mashhad (44%), 39% in the other urban and rural areas of Khorasan province and 17% were migrants of other provinces that have been in Mashhad within the past five years and had decided to live in Mashhad for their whole life. Nine women had two or more children at the time of interview.

**Data collection procedure**

The main data collection method was face-to-face, semi-structured interview with participants. Before starting the interviews, the participants were appreciated for taking part in the study and confidentiality and anonymity of the interview process were emphasized. Then women were asked about their decision for having a child and being satisfied with the timing of the birth of their first child. In order to assess the social network influence, at first, women were asked about the fertility beliefs and behaviors in the family they grew up in, then it was discussed when they started to think about having a child for the first time and how these ideas developed later on, especially after marriage. The main emphasis of the interview was eliciting of "for and against ideas", communication and negotiation with relevant others and the way that it affects couples’ decision about having the first child. 

Participants could explain in their own words about their important referred individuals, how the referees played a role in their intention with regard to having their first child and how they and their spouses responded to and complied with those relevant others. Probing questions were asked based on the answers of participants. The interviews were scheduled at a place convenient for each of the participants. All interviews were conducted between August 2011 and April 2012 by the first author who is a midwife researcher with a good research experience in the family planning settings. The time and duration of interviews were determined in a mutually agreed manner. Each session of the face to face interview took average between 80 to120 minutes. For nearly half of the participants the second and for one participant the third interview was conducted. 

**Data management and analysis**

All interviews were recorded electronically, fully transcribed verbatim for data analysis. Transcripts were read repeatedly and tapes listened frequently to achieve immersion, get an insight to whole interview and obtain the sense of the whole, as stated by Hsieh and Shanon (26). Summaries were written throughout the analysis process. Transcripts were coded and analyzed using MAXqda software package, Version 2. Conventional content analysis technique that involves in-depth interpretation of the underlying meaning of the text and condensing data without losing its quality was used (27). 

Events, actions, explanations and perceptions were identified and coded. The codes were grouped into subcategories and categories (Table I). The analysis was discussed among the research team members. Analysis was verified via member validation of cods (28). During this period the codes and emerging categories were discussed with the project supervisor (the second author), who is an experienced qualitative researcher, in order to verify the interpretations.

**Ethical considerations**

The study was approved by Research Ethics Committee, Mashhad University of Medical Sciences, Mashhad. All participants signed the informed consent form and were assured that confidentiality and anonymity will be maintained. They could withdraw from the study at any time without prejudice to their management.

## Results

Data analysis demonstrated four major categories about social network’s influence on couples’ intention to have their first child including 1) perception of fertility relevant social network, 2) occurrence of various types of social influence 3) subjective judgment of the benefits of social network and its fitness to personal life, and 4) couples’ interaction with social network. 

**Perception of fertility relevant social network**

The sphere of social network extensively varied for different participants. Social network members included relatives, mostly parents and sisters, the mothers and sister-in-laws as well as the cousins especially those of the same age, and friends, colleagues and neighbors in some participants. 

A significant network partner meant for most of the participants as somebody who had more experience and closer emotional relationship or was more convenient in mutual relationships or had physical closeness and frequent contacts. 

The more significant fertility relevant social network varied based on educational level and the socio-economic status of the participants. Educated and employed participants considered their experienced and expert relatives, colleagues, friends particularly those with expertise in medicine and health as ‘higher significant’ than persons with traditional beliefs. The expression of one of participants reveals this point: *“One of my good colleagues influenced me with regard to my work, my life and my childbearing. I prefer the advice of such persons.” (W2, 29 years, para 2) *Whereas, housewives and lower educated participants communicated more frequently with the persons with closer familial relationships and those in closer physical relations that were more convenient to them to talk, such as their own mothers and sisters, mothers and sister-in-laws and in few cases the neighbors. One of these participants said: *“I only knew my relatives and usually communicate with them and also two of my neighbors…. I only talked to them in such issues, when it was needed.” (W6, 34 years, para 3)*

A small number of participants mentioned health care workers as significant references in this regard. Nearly three fourth of the participants mentioned no contact with reproductive health care providers after the marriage and before their first pregnancy. *“I haven’t seen that people go to the health centers before their first pregnancy, I think, they go there when they want contraceptives.” (W5, 46 years, para 3). *A Midwife in this regard commented: “*Some of the clients come to receive pre-conception care before their first pregnancy, but their number is not remarkable, in most cases the first referral to the health centers is when they are pregnant.” (Midwife 3, 40 years, unmarried)*

**Occurrence of various types of social influence**

Social issues that affected the couples’ intention to have their first child or to be a parent categorized as “social lessons learned”, “social strains” and “social support”. 

**Social lessons learned**

Making decision on having a child and its time was influenced by couples’ social learning, which was occurred via paying attention to explicit experiences in family of origin and other relevant network members. The following expression reveals this concept: *“Everybody around me had at least one baby. I think it is natural, as everybody wants at least one child” (W11, age 31, Para 2).*

A majority of women stated that the opinions of their parents and relatives about the suitable age of marriage and first childbirth contributed in forming their attitude and belief about the childbearing and the age of its initiation. One of the participants in this regard said: *“When the parents believe that childbearing in a special age is right; they try to convey such beliefs to their offspring” (W12, age 29, Para 2). *

A few women pointed out that their parents who had experienced childbearing in a lower age and had faced some challenges advised them not to act like them. *“My mom gave me birth at the age of 13, she always advised us to have our first child after our graduation from the university. Now I, as a mother, wish my girl to marry at 24-25 and give her birth 2-3 years later” (W8, age 46, Para 1).*

The important sources of social learning with respect to the time of the first childbearing included the mothers, mother-in-laws, sisters, sister-in-laws, friends and colleagues of the same age or educational level, either with satisfactory or unsatisfactory birth experiences. Such attitudes were shaped in couples’ mind via observing network’s life events and the people’s reactions to such events, or through verbal communications and receiving comments from the others about them. 

**Social strains**

Most of the participants condemned the habits of some people who frequently asked about their childbearing. Such issue was frequently pointed out by the participants: *“There is no life issue as fertility, about which people are so eager to ask and interfere. Whenever they see a new married woman, they immediately ask “are you pregnant, why not? Why you don’t think of that?” (W11, age 31, Para 2).*

Women of older ages at marriage were more persuaded by their network to have their first child as soon as possible. The families of spouses were usually more sensitive to the ability of their daughter-in-law for being fertile. Some of network members had more power to enforce their expectations to the couples. “*It was only 8 month past of our marriage,… my mother in law had said to my husband that I might be hereditarily infertile, as I have an aunt who was infertile” (W10, 33 years, para2).*

Some advised the couples as they were worried about them and their life: *“We had no child for 6 years.… My family also reminded me frequently to decide for having a child and not to permit any dilemma to be occurred.” (W7, age 43, Para 1).*

Sometimes couples strained by the colleagues and friends to change their beliefs and intentions. This pressure was stronger when couples had no children for a long time because of voluntary postponement of the first childbirth or due to other reasons such as sub-fertility or infertility. *“During these 5 years that I had no child voluntarily… Some bodies thought that we are probably infertile. Sometimes, they had a sense of sympathy with me and suggested me meeting an expert doctor whose they know.” (W2, 29 years, para 2).*

Some of the participants in this study stated that for the purpose of avoiding the labeling and also to control the psychological pressure surrounded the childless couples, their parents persuaded them to have at least their first child soon. The mother of a participant said: *“I said to her (my daughter) if you don’t have a child, nobody believes that you’ve decided to do so yourself; the people will circulate gossip about you.” (Grandmother, age 51, Para 3). *In this study the majority of couples were highly suggested by their relatives not to prevent pregnancy early in their marital life; as they believed that it may cause infertility. 

Additionally, they were recommended not to use any kind of contraceptive methods even the condom or natural ones. These sorts of strains usually had deep influence on the couples too. One of the midwives in this relation commented: *“My relatives advised me not to have any contraception before having the first child, but I did so for three* *years, thereafter* *my pregnancy didn’t happen until a prolonged course of treatment. So I believe this and always said it to my clients.” (Midwife2, 47 years, para 2). *

A majority of participants mentioned a pressure from their network for being cautious about not becoming pregnant during the engagement period. Engagement is a period of time between the couples’ legal marriage and starting formal marital life in a “joint home”. This period varies from few days to a few years, in which each of the spouses stayed with their parents. Pregnancy in engagement period is associated with blame from the other people and some forms of punishment from the couples’ parents. A woman who had become pregnant in this period said: *“Everybody blamed me, even after going to our joint* *home and after giving birth to my child.” (W1, 43 years, para 3).*

All midwives mentioned frequently visiting such women that faced a great deal of problems. One of the midwives participated in this study commented: *“I approach a girl who became pregnant in engagement period. Yet, after years, her parents do not talk with her. They gave her a little dowry; they don’t pay any attention to her.” (Midwife1, 43years, para1).*

There were two couples who perceived very little negative pressure in case of pregnancy occurrence in engagement period. Their families were less sensitive to this situation and the couples could cope much easier with this concern due to having good family support.

**Social support**

The findings of this study demonstrated that having social support either as informal or formal, could influence the decision of couples about their first childbirth. Informal types of social supports were more frequent compared to the formal ones. Some of the participants indicated that their parents and parents-in-law provided various forms of informal support to them including promising to help in taking care of the child. 

They also provided emotional and financial support for the couples, which promoted the couples’ intention for having their first child in desired time especially in cases that the wife was employee or student. While a lack of support often caused to postpone having the first child. Siblings and friends also were other sources of emotional support and advice for couples in child caring and providing some necessary helps to them. 

Formal social support was mainly delivered by health workers that provided their services for new married couples in a few occasions. Most of couples had participated in pre-marital classes in which a brief education was given to them about the family planning and other available reproductive health services. They also supported couples by providing some helpful information, counseling services and providing them with family planning methods in request of couples. In this study there were just few women who used such services before having their first child. Indeed the pre-marital classes did not often give them enough insight about the services delivered by the health care system. One of the midwives in this regard indicated: *“In urban districts, primary health system coverage is not so good and most of newly married couples are not familiar with this system and so don’t use it.” (Family health provider1, 28years, para1).*

Due to receiving no instruction or just little information about sexual issues and family planning before the marriage, most of the newly married couples perceived a great need to such information and help during their engagement period. At the same time, they perceived some barriers and constrain to use the available family planning services, as their families often expected them not to have sexual relationship in this period. 

Some couples that hide their sexual relationships might attend the physician’s or midwives’ offices to take emergency contraceptives or to get help when they were in doubt to be pregnant unintentionally. One Midwife in this relation commented: *“There are some women that come to us in their engagement period and ask for contraception, and we would provide them … Some of them come only when they are pregnant and cannot hide it longer.” (Midwife1, 43years, para1)*

**Subjective judgment about the benefits of social network and its fitness to personal life**

Based on the study findings, there were various types of social influence perceived by couples, which changed the pattern of their fertility decisions on the time of having the first child.

 All of the participants in this study emphasized that they learnt lessons through communication with others and affected by social strains or social supports and thought and judged about the extent of their relevance and fitness to their own life and evaluated such communications: *“It was not as such that we don’t pay attention to our relatives’ views. We judged what talks are truthful, I mean, what option does really benefit us. We accepted it if it was fit with our goals and ambitions (W2, 29 years, para2). *

Participants’ perception regarding fertility could be influenced by their positive or negative evaluation of fertility experiences of family. Couples who evaluated the experiences of their mothers and other network members as satisfying were interested to start childbearing in a similar way to their parents and those with negative experiences in their network, tried to avoid such experiences. 

If they assumed their network members’ advice are not adjusted and fit to their wishes and life plans, they refused it. In fact some of the participants believed that their network experiences or their advice for having a child interferes with the successful completion of their academic studies and finding secure and stable jobs. Some of them stated that they wish to fulfill their youth ambitions and enjoy from their marital relationships and some mentioned other causes for not accepting their network beliefs and advice. One participant in this regard elaborated: *“Everybody around, had their first child early in their young age, but we wanted to promote. Indeed, my husband wanted to get job stability. It was not suitable for us. We needed to postpone bearing our first child several years after the marriage.” (W3, 35years, para1).*

Noticeably, it was easier for participants to accept the opinions and advice of network members with similar educational and employment status and satisfying childbearing experience. The suggestions and advice of such person was better accepted by the participants compare with those from the close family members’ with different life conditions. *“I would accept the talks of the persons whose level of understanding and condition is similar to me, as they are able to understand me much better. I could certainly use the experiences of such people.” (W11, 34 years, para 2). *

**Couples’ interaction to social network**

According to the findings of study, the patterns of participants’ responses to social powers were different. Some stated that social network has an obvious impact and some others stated that it has little or very little effects on their plan for starting the childbearing. 

When the networks’ views were not consistent with couples’ mutual decision, they tried to weight the social strains alongside with their level of empowerment to refuse or to go along with. The couples’ level of empowerment was an important factor regarding acceptance or refusing the network’s influences. It seems that the couples who resisted against social strains had higher control on their individual life. Some women stated that they always resist against the network’s influences when they suppose it is not adjusted to their life plan. A woman said: *“When I make a decision, I persist on that. Even their gossip, and labeling us as infertile has no influence on me and my husband.” (W17, 32 years, para1).*

Some others stated that because of powerful social strains and having low empowerment for resisting against it, they decided not to prevent pregnancy in spite of being unprepared for having a child. In fact, they found themselves obliged to comply networks’ views. "*I didn’t like to bring a child so soon. But my mother frequently asked me and tried to convince me… So I became pregnant 11 month after my marriage.” (W20, 26years, para1).*

Some participants’ stated that their views about the significance of the others’ opinion have been changed. For example if couples found that a network partner was not able to comprehend their condition or emphasized on some expectations that was not fit to their life, the couples’ evaluation of network opinions was changed to a situation of ‘less or not significant’. Health care providers also moved into the position of ‘little or no significance’ when they could not understand the fertility needs and situation of a couple. In such a circumstances, the couples relied on other non-expert people who were reliable from their viewpoint and were able to understand their life condition. 

The expression of one midwife reveals this meaning: “*She was seeking help to abort her fetus…. I advised her not to do so… but she didn’t come back here for a long time until a few days ago, and …, I realized that my talks had no effect on her decision. She said that she received some instructions from one of her friends to abort by using herbal medicine stuff and has aborted her fetus” (Midwife3, 42 years, unmarried)*.

**Table I:** Examples of codes, subcategories and categories of the social network influence

**Meaning unit**	**Condensed meaning unit**	**Code**	**Subcategory**	**Category**
				Occurrence of various types of social influence
			Social lessons learned	
Everybody around me had at least one baby. I think it is normal.	Observing network’s life and its acceptance	Learning through observation		
My mother always said to me and my sister not to do as she did.	Receiving information by the family	Network’s impact on learning		
			Social support	
My family always said to me that no matter you bring a child, we help you in caring him.	Family promising to help in taking care of the child	Promising for support		
When I want to go to work, what I should do with my child? There is no suitable nursery around us.	Lack of suitable nursery around	Not enough social support		
			Social strains	
Our neighbour frequently blamed my wife for not having a child.	Blaming the couples	Interfering		
Somebodies thought that we are infertile. …, They even had a sense of sympathy with me and tried to help me with their advice.	Labelling as infertile (Being sympathetic and giving advice)	Labelling Lay persons' sympathy		

## Discussion

The aim of this study was to explore the influence of social network on individuals’ decision about having the first child. Data analysis showed that personal relationships had a salient influence on couples’ experience of having their first child. Although the studies of social network has provided valuable insights towards the way of forming and changing participants’ attitude and intentions about the first childbirth, but applying qualitative research in this study allowed exploring the complex pattern of relationships within social network and its influence on starting the reproduction, which is something that has received less attention in the previous studies. 

The social network’s influence as experienced by participants in this study consisted of four main categories: 1) perceived fertility relevant social network, 2) occurrence of various types of social influence, 3) subjective judgment about the benefits of social network and its fitness to personal life and 4) couples’ interaction with social network. 

In this study, fertility relevant social network mostly included the referees who have been more experienced or expert, more convenience in relationships and the ones who have had more emotional and physical closeness. Such characteristics varied for women of different socio economic backgrounds, a finding that has not been reported in the previous studies. In the study of Keim *et al*, the important network mostly includes the family members, close friends and acquaintances. The ‘important person” in their study was the one who has had emotional and supportive relationship, intimacy and frequency of contacts with the participants (6). Steenhof and Liefbroer discussed the intergenerational age at entering into parenthood (20).

This study showed various types of social influence that affected couples from the phase of their attitude formation to active phase of decision making about their first childbirth. The types of social influence in this study were categorized as social lessons learned, social strains and social supports that were in some extent similar to the findings of Bernardi in Italy and Keim *et al* in Germany on family formation and reproduction (6, 17). 

The other point is that the type of issues and content of social messages perceived by couples in the current study was often different from the previous studies that have been conducted in the societies with different context of Iranian society. It seems that multiple social factors have contributed to forming primary perceptions and attitudes during the life period. Social strains and social supports functioned more after the marriage when the couples were involved in childbearing decisions. This study showed that strong power of social network affect the formation of couples’ intention for having a child and regulating its time that is consistent to findings of Bernardi (17). The previous studies described the persons and couples as socialized actors embedded in a network of informal interactions with relatives and peers. These studies suggested that the researchers should consider the social mechanisms in their models (29).

The findings of this study also showed that new married women have some unmet needs to family planning and reproductive health services that originate from social constrains for using available services. It is needed to provide services in a new way and finding appropriate solutions based on social and cultural norms. The majority of newly married women in this study reported having a fear of contraceptive use, which mostly was due to misunderstanding of other people’s experiences in this regard. They feared from infertility following using contraceptive methods soon after marriage. At the same time, they perceived infertility as a sever threat for their marital life. Such fear was reported frequently and with more severity by women. The study of Baghiani Moghadam *et al* showed the higher psychological effect of infertility in Iranian women compared to men (30). Thus, some of participants wanted to have a child sooner. 

This study also demonstrated the importance of participants’ subjective evaluation of the fitness of the network partners’ experiences, beliefs and advice for their life. If the outcome of such evaluations was positive or consistent with couples’ decision, the network information was most likely applied. Other studies have shown that the people’s satisfying or unsatisfying experience in their family of origin determines whether they attempt to recapture the former experience while constructs a family (31). As the study of Keim demonstrated the experience of networks’ member with fertility at younger age dose not routinely persuade participants to start their childbearing soon (6). 

Conversely, it sometimes encourages couples to postpone their first childbirth. It happens especially when the participants believe that having a child is incompatible with their life plan. The study of Liefbroer* et al* also showed that the women evaluate how other family lives is and to what extent the others’ condition is comparable to theirs (31). In this study the majority of participants’ attitude towards the use of birth control methods early in marital life was formed by parents and social network members recognized by the couples. This finding was congruent with the findings of Anderton (32). Sometimes the fear of infertility or breaking down the newly formed marital life influenced couples’ fertility decisions and allowed other rational judgments to be overcome. 

This was an important finding of this research which has not been reported in the previous studies. This study also illustrated the importance of participants’ subjective evaluation about the power of such social factors and their perceived ability to resist against it. In fact, when couples’ subjective analysis and judgment resulted in incompatibility of network partners’ expectations, it could lead to two patterns of interaction including complying or refusing that. They sometimes perceived the social strains as strong or weak. Also they might evaluate themselves as empowered or powerless. 

It seems that their response to such social strains depends on their perceived power and their perception on the level of their empowerment to resist against it. This finding is seemed original and has not been reported in previous studies. In addition, this study showed the changes in network relationships towards increasing communications with friends and colleagues who were more similar to the couples in terms of life conditions and socio-cultural and socioeconomic status. The changes also happened in the degree of compliance with the referees in relation to the issue of having a child. The study of Keim *et al* also showed resistance against social powers or changing the network structure for the purpose of overcoming the network’s effect (6). The limitation of this study was that women were expected to recall events which had been occurred in some cases at a long period ago; although the participants believed that they never forget their experiences of the first childbirth. 

## Conclusion

The findings of this study showed the important role of social network on couples’ intention to have their first child. The findings particularly contribute to the little evidence on social networks and its role in forming and changing the fertility intentions. The findings also showed the especial needs of newly married couples to reproductive health information, counseling and services that should be noticed and met in health care delivery system. According to the study findings, women’s empowerment was the essential strategy that enabled them to make informed and freely decision about starting their fertility and overcoming any social power which is not fit to their life plan. It also gave the women the choice to select their beneficial personal relationships.
